# Confirmatory factor analysis of the Korean version of the short-form McGill pain questionnaire with chronic pain patients: a comparison of alternative models

**DOI:** 10.1186/s12955-014-0195-z

**Published:** 2015-02-07

**Authors:** Sun Ah Choi, ChongNak Son, Jang-Han Lee, Sungkun Cho

**Affiliations:** Department of Psychology, Chung-Ang University, 84 Heukseok-ro, Dongjak-gu, Seoul, 156-756 Korea; Department of Psychology, Chonbuk National University, Deokjin-dong 1ga, Deokjin-gu, Jeonju-si, Jeollabuk-do 561-756 Korea; Department of Psychology, Chungnam National University, 99 Daehak-ro, Yuseong-gu, Daejeon, 305-764 Korea

**Keywords:** Short-form McGill pain questionnaire, Chronic pain, Korean, Confirmatory factor analysis

## Abstract

**Background:**

The Short Form of the McGill Pain Questionnaire (SF-MPQ) is the most widely used assessment of the quality and intensity of pain. In previous validation studies, the factor structure of the SF-MPQ varied widely from various two-factor structures to a five-factor structure, although research on the SF-MPQ quite consistently supports its two-factor structure (i.e., sensory and affective) across different countries and languages. In Korea, the results of exploratory factor analysis of a Korea version of SF-MPQ (KSF-MPQ) showed 2-factor structure consisting of ‘sensory’ and ‘affective’ excluding two items such as splitting and heavy. As an attempt to further validate the KSF-MPQ, the purpose of this study was to confirm whether the KSF-MPQ model is an appropriate model for chronic pain patients in Korea by comparing several alternative models of the SF-MPQ.

**Findings:**

A total of 150 chronic pain patients seeking treatment in Seoul, Korea, participated and completed the KSF-MPQ. Confirmatory factor analysis was conducted to evaluate the adequacy of the KSF-MPQ model and several alternative models. The results indicated that the adjusted KSF-MPQ model showed the best fit to the data among the models in chronic pain patients in Korea.

**Conclusions:**

The results showed the KSF-MPQ is cross-culturally equivalent to the original questionnaire. Thus, the KSF-MPQ is valid measurement for assessing the quality and intensity of pain to chronic pain patients and may be helpful in clinical and research settings in Korea.

## Background

Chronic pain is defined as pain that persists for ≥ 3 months [1,2] and usually does not respond to conventional treatment or surgery [3]. As a result of this long-lasting pain, many chronic pain patients (CPPs) face restrictions in their daily activities [4]. For example, the fatigue and mobility limitations accompanying chronic pain can lead to a deterioration of physical function, possibly resulting in disability [5,6]. Additionally, CPPs are likely to have psychological problems (e.g., anxiety, depression, sleep disorders), which often lead to substance abuse and even suicide [7,8]. In such situations, the proper measurement of the quality and intensity of painful experiences would be useful for formulating a plan of treatment and predicting its outcome [9].

The Short Form of the McGill Pain Questionnaire (SF-MPQ) is the most widely used assessment of the quality and intensity of pain [10]. The SF-MPQ is an abbreviated form of the McGill Pain Questionnaire [11] and is used in medical settings in place of the long-form questionnaire for pragmatic reasons. The SF-MPQ purports to measure sensory and affective pain (referred herein as the Melzack model) and has been widely validated in many languages and countries. The sensory category (e.g., shooting, sharp) focuses on the nociceptive pain experience, and the affective category (e.g., tiring-exhausting, fearful) focuses on the emotional component of nociceptive pain [12]. In previous validation studies, the factor structure of the SF-MPQ varied widely from various two-factor structures to a five-factor structure, although research on the SF-MPQ quite consistently supports its two-factor structure (i.e., sensory, affective) across different countries and languages [12].

For example, the exploratory factor analysis (EFA) of a Korean version of the SF-MPQ (KSF-MPQ) has been performed in CPPs [13]. The results yielded a two-factor structure consisting of ‘sensory’ and ‘affective’ factors, excluding the two items referring to ‘heavy’ and ‘splitting’ (referred herein as the KSF-MPQ model) [13]. Wright et al. [10] performed confirmatory factor analysis (CFA) for patients with chronic back pain. To meet the criteria of the model fit indices, they set item 6 (gnawing) as an affective instead of sensory category and correlated four sets of error terms. They then obtained a two-factor structure consisting of ‘sensory’ and ‘affective’ factors (referred herein as the Wright model). Shin et al. [14] performed EFA for Asian-American cancer patients and obtained a two-factor structure that differs from the Melzack model (referred herein as the Shin model). Burckhardt and Bjelle [15] performed EFA on a Swedish version of the SF-MPQ for female patients with either fibromyalgia or rheumatoid arthritis. The EFA produced three factors: the sensory category was divided into acute-sensory and chronic-sensory, and the affective category was retained (referred herein as the Burckhardt model). Cassisi et al. [16] performed EFA for African-Americans and European-Americans with chronic pain, obtaining a five-factor solution for African-American patients (referred herein as the Cassisi A model) and a four-factor solution for European-American patients (referred herein as the Cassisi B model).

To examine the possibility of utilizing the KSF-MPQ in medical and research settings, further validation of the KSF-MPQ is necessary. As previous studies have shown different factor structures of the SF-MPQ across countries or cultures [10], it is especially important to examine its appropriateness for Korea. Thus, this study aimed to confirm whether the KSF-MPQ model is an appropriate model for CPPs in Korea by comparing several alternative models of the SF-MPQ using CFA.

## Methods

### Participants

A total of 157 CPPs visiting a pain center in Seoul, Korea, participated in this study. The inclusion criterion for the study was pain duration of at least 3 months. The patients (n = 7) who had experienced pain for < 3 months were excluded, leaving 150 eligible patients. Table 1 presents the demographic characteristics of the participants. All data were collected and analyzed after obtaining approval by the Institutional Review Board (Seoul St. Mary’s Hospital) and informed consent from the participants.

**Table 1:** **Demographic characteristics of the sample**

**Variable**	**Sample (N = 150)**
Age (years)	
M	41.9
SD	12.8
Sex (%)	
Male	41.3
Female	58.7
Marital status (%)	
Married	57.3
Non-married	42.7
Educational status (%)	
≥ High school	95.3
Pain duration (months)	
Median	37
Range	3-240
Taking pain-related medication (%)	60
Pain category (%)	
≥2 sites	48.7
Lower back	26.0
Head	16.9
Shoulder(s)	14.3
Leg(s)	11.7
Others	31.1

### Measures

Quality and intensity of pain were measured by the KSF-MPQ. The KSF-MPQ consists of 17 items, 15 of which are adjectives from the 11 sensory and 4 affective categories that are rated on a 4-point intensity scale from 0 (not at all) to 3 (all the time). The other two items assess overall pain intensity: the Present Pain Intensity (PPI), which is rated on an intensity scale from 0 (no pain) to 5 (excruciating), and a Verbal Analogue Scale (VAS), which consists of a 10-cm line on which pain is rated between 0 (no pain) and 10 (worst possible pain). The PPI and VAS were excluded in the present analysis.

### Statistical analyses

Data for the statistical analyses were examined using SPSS 17.0 and Amos 20.0 for Windows. CFA was conducted to evaluate the adequacy of the models. Because a factor consisting of a single item cannot be analyzed in CFA, two factors of the Cassisi A model were excluded. Thus, three out of five factors were analyzed in CFA. The indices used to evaluate model fit in CFA include the root-mean square error of approximation (RMSEA), comparative fit index (CFI), normed fit index (NFI), Tucker-Lewis Index (TLI), Akaike information criterion (AIC), and Bayesian information criterion (BIC). RMSEA values of < .05 indicate a good fit to the data, values between .05 and .08 an acceptable fit, values between .08 and .10 a marginal fit, and values > .10 a poor fit [17]. For the CFI, NFI, and TLI, values > .90 indicate a good fit to the data [18]. For the AIC and BIC, smaller values indicate a better fitting model.

## Results

To obtain an adequate model for CPPs in Korea, CFA with maximum-likelihood estimation was conducted. All models, except the Wright model, were adjusted based on a combination of logical and empirical indicators (i.e., modification indices) guiding path additions. Tables 2 and 3 presents summary of the models and the model fit indices for the models of the SF-MPQ, respectively. The adjusted KSF-MPQ model showed a good model fit for the CFI, NFI, TLI, and RMSEA, and had the lowest values for the AIC and BIC among the models. A single-factor model produced the worst fit to the data. The Wright, adjusted Burckhardt, and adjusted Melzack models displayed a marginal model fit for the RMSEA and a good model fit for the CFI but an inadequate model fit for the NFI. Also, the adjusted Burckhardt and adjusted Melzack models showed a good model fit for the TLI but the Wright model did not. The adjusted Cassisi B model displayed a good model fit for the CFI and TLI, a marginal model fit for the RMSEA, and a poor model fit for the NFI. The adjusted Cassisi A and adjusted Shin models displayed a poor model fit for the CFI, NFI, TLI, and RMSEA. Thus, among the models studied, the adjusted KSF-MPQ provided the best model fit to the data for CPPs in Korea. The internal consistency for the total, sensory, and affective scale scores of the KSF-MPQ were Cronbach’s α = .93, .90, .91, respectively.

**Table 2:** **Summary of each model’s description**

**Item/Models**	**KSF-MPQ** ^**1**^	**Single-factor**	**Melzack**	**Wright**	**Shin**	**Cassisi A**	**Cassisi B**	**Burckhardt**
1. Throbbing	S	M	S	S	SF1		CF4	A-S
2. Shooting	S	M	S	S	SF1		CF2	A-S
3. Stabbing	S	M	S	S	SF1	CF3	CF2	A-S
4. Sharp	S	M	S	S	SF1	CF2	CF2	A-S
5. Cramping	S	M	S	S	SF2	CF2	CF4	A-S
6. Gnawing	S	M	S	A	SF2	CF2	CF1	C-S
7. Hot-burning	S	M	S	S	SF2	CF3	CF4	A-S
8. Aching	S	M	S	S		CF3	CF1	C-S
9. Heavy	·	M	S	S	SF1	CF2	CF1	C-S
10. Tender	S	M	S	S	SF2	CF3	CF4	C-S
11. Splitting		M	S	S	SF1	CF1	CF3	A-S
12. Tiring- exhausting	A	M	A	A	SF1	CF2	CF1	A
13. Sickening	A	M	A	A	·	CF1	CF3	A
14. Fearful	A	M	A	A	SF1	CF1	CF3	A
15. Punishing-cruel	A	M	A	A	SF1	CF1	CF3	A
Number of item	13	15	15	15	13	13	15	15

^1^KSF-MPQ: Korean version of Short-Form McGill Pain Questionnaire, MSF-MPQ: Modified Short-Form McGill Pain Questionnaire, M: SF-MPQ, S: sensory, A: affective, S-A: sensory-affective, SF1: Shin factor 1, SF2: Shin factor 2, CF1: Cassisi factor 1, CF2: Cassisi factor 2, CF3: Cassisi factor 3, CF4: Cassisi factor 4, A-S: acute-sensory, C-S: chronic-sensory, ·: not included. Table 1 was referred from research by Mason et al. [12]; A figure depicting each model will be provided upon request.

**Table 3:** **Model fit indices for SF-MPQ**

**Model**	***x*** ^**2**^ **(** ***df*** **)**	**RMSEA**	**CFI**	**NFI**	**TLI**	**AIC**	**BIC**
KSF-MPQ^1^	126.88 (64)	.08	.95	.90	.94	180.88	262.17
Adjusted KSF-MPQ^2^	101.86 (63)	.06	.97	.92	.96	157.86	242.16
Single-factor	343.94 (90)	.14	.81	.76	.78	403.94	494.26
Adjusted Single-factor^2^	319.03 (89)	.13	.83	.78	.80	381.03	474.36
Melzack	202.94 (89)	.09	.92	.86	.90	264.94	358.27
Adjusted Melzack^2^	180.34 (88)	.08	.93	.88	.92	244.34	340.68
Wright^3^	204.11 (85)	.10	.91	.86	.89	274.11	379.49
Shin	259.62 (64)	.14	.83	.79	.79	313.62	394.90
Adjusted Shin^4^	198.69 (63)	.12	.88	.84	.85	254.69	338.99
Cassisi A	195.57 (62)	.12	.88	.85	.86	253.57	340.88
Adjusted Cassisi A^5^	189.40 (61)	.12	.89	.85	.86	249.40	339.72
Cassisi B	212.75 (84)	.10	.91	.85	.88	284.75	393.13
Adjusted Cassisi B^2^	191.42 (83)	.09	.92	.87	.90	265.42	376.81
Burckhardt	200.01 (87)	.09	.92	.86	.90	266.01	365.36
Adjusted Burckhardt^2^	176.14 (86)	.08	.93	.88	.92	244.14	346.50

^1^KSF-MPQ: Korean version of Short-Form McGill Pain Questionnaire; ^2^Covariance between error terms for items 1, 8; ^3^The Wright model basically includes specified covariance between error terms for items 2, 3; 3, 4; 2, 4; and 8, 12 and thus, the Wright model was not adjusted in the present study; ^4^Covariance between error terms for items 14, 15; ^5^Covariance between error terms for items 12, 13; RMSEA: Root-Mean Square Error of Approximation; CFI: Comparative Fit Index; NFI: Normed Fit Index; TLI: Tucker-Lewis Index; AIC: Akaike Information Criteria; BIC: Bayesian Information Criteria.

## Discussion

Findings indicated that the (adjusted) KSF-MPQ model provides the best fit for CPPs in Korea, which is consistent with the EFA result of the KSF-MPQ in Korea [13]. Although the KSF-MPQ does not contain two items (i.e., heavy, splitting), it was fundamentally consistent with the original Melzack model in terms of its components (i.e., sensory, affective pain). These two items were excluded due to low factor loadings in the prior study [13]. One possible explanation is that they are likely to best suit patients suffering from pain in a specific site. For example, ‘splitting’ tends to be used by patients with severe headaches, and ‘heavy’ tends to be used by patients with lower back pain. Moreover, given that these words are not frequently used in Korea, the factor structure of the SF-MPQ may be different in Korea. Future studies should replicate that those two items should be dropped for a Korean sample.

This study compared several models for CPPs in Korea to identify the most adequate model and suggests that the KSF-MPQ is suitable for assessing the quality and intensity of pain. These results showed that the KSF-MPQ is cross-culturally equivalent to the original questionnaire. Based on the results of the present study, the KSF-MPQ may be a useful clinical tool for assessing patients’ current state and treatment planning. Using the KSF-MPQ, both patients and health professionals can monitor the patients’ condition more closely and take appropriate action [19].

Nevertheless, this study has an important limitation. The patients who participated in this study were CPPs who reported pain in different areas and may not be generalizable to patients with specific pain site(s). Further study needs to be done with large numbers of patients with specific pain site(s).

## Conclusions

The KSF-MPQ is a valid questionnaire for assessing the quality and intensity of pain experienced by CPPs in Korea. Thus, the KSF-MPQ may be a useful tool for evaluating the pain experience of CPPs and may be helpful in clinical and research settings in Korea.

## Abbreviations

CPP: Chronic Pain Patient
SF-MPQ: Short-Form McGill Pain Questionnaire
KSF-MPQ: Korean version of Short-Form McGill Pain Questionnaire
EFA: Exploratory Factor Analysis
CFA: Confirmatory Factor Analysis
RMSEA: Root-Mean Square Error of Approximation
CFI: Comparative fit index
NFI: Normed fit index
TLI: Tucker-Lewis Index
AIC: Akaike information criterion
BIC: Bayesian information criterion
